# Association of lipoproteins and thyroid hormones with cognitive dysfunction in patients with systemic lupus erythematosus

**DOI:** 10.1186/s41927-021-00190-7

**Published:** 2021-06-09

**Authors:** Li Lu, Wei Kong, Kangxing Zhou, Jinglei Chen, Yayi Hou, Huan Dou, Jun Liang

**Affiliations:** 1 Department of Rheumatology and Immunology, Nanjing Drum Tower Hospital, The Affiliated Hospital of Nanjing University Medical School, Nanjing, 210008 PR China; 2 The State Key Laboratory of Pharmaceutical Biotechnology, Division of Immunology, Medical School, Nanjing University, Nanjing, 210093 PR China; 3 Jiangsu Key Laboratory of Molecular Medicine, Nanjing, 210093 PR China

**Keywords:** Systemic lupus erythematosus (SLE), Cognitive dysfunction, Free triiodothyronine (F-T3), Free thyroxine (F-T4), Lipoprotein

## Abstract

**Background:**

Neuropsychiatric manifestations occur in up to 75% of adult systemic lupus erythematosus (SLE) patients and are one of the major causes of death in SLE patients. Cognitive dysfunction is a typical clinical feature of neuropsychiatric SLE (NPSLE), which seriously affects the quality of life of patients. Dyslipidaemia and thyroid symptoms, which are prevalent in SLE patients, have both been related to neuropsychiatric disturbances, including significant psychiatric and cognitive disturbances. This study aimed to investigate whether cognitive dysfunction in patients with SLE was related to the expression of serum thyroid hormone and lipoprotein levels.

**Methods:**

A total of 121 patients with SLE and 65 healthy controls (HCs) at Nanjing Drum Tower Hospital completed a cognitive function test, and 81 SLE patients were divided into a high-cognition (*n* = 33) group and a low-cognition group (*n* = 48). The clinical and laboratory characteristics of the patients were compared; moreover, correlations between serum HDL-C, LDL-C, F-T3 and F-T4 levels and cognitive function were analysed. Serum levels of APOE, APOA1, IGF-1, and IGFBP7 in 81 patients were detected by ELISA, and the correlation between these four proteins and cognition was analysed separately.

**Results:**

The patients with SLE with abnormal cognitive function were less educated than the HCs. For low-cognition patients, the levels of albumin, F-T3 (*P* <  0.05) and F-T4 decreased, while D-dimer, anti-dsDNA antibody, and IgM levels increased. Serum F-T3 and F-T4 levels positively correlated with cognition. Furthermore, serum protein levels of APOE and APOA1 showed no difference between the high- and low-cognition groups. However, the serum APOE levels were negatively correlated with line orientation scores, and APOA1 levels were positively correlated with coding scores.

**Conclusions:**

Serum F-T3 and F-T4 levels were both positively correlated with four indexes of cognition (language was the exception), while serum APOE levels were negatively correlated with line orientation scores, APOA1 levels were positively correlated with coding scores, and IGFBP7 levels were negatively correlated with figure copy scores. These results demonstrated that F-T3 and F-T4 might be clinical biomarkers of cognitive dysfunction in SLE.

**Supplementary Information:**

The online version contains supplementary material available at 10.1186/s41927-021-00190-7.

## Introduction

Systemic lupus erythematosus (SLE) is a chronic systemic autoimmune disease characterized by autoantibody production and immune complex deposition, culminating in destructive injuries to multiple organs, including central nervous system (CNS) [1, 2]. Neuropsychiatric SLE (NPSLE) is a manifestation of SLE associated with severe neuropsychiatric (NP) syndromes, including various neurological and psychiatric features [3]. Several studies have estimated that up to 75% of patients with SLE experience NP manifestations [4, 5], including mood disorders, confusion, headache, and cognitive dysfunction, which significantly degrade quality of life and affect the survival of patients [6]. However, the underlying disease mechanisms remain largely unknown, as NP symptoms are nonspecific, clinically validated biomarkers for diagnosis are nonexistent, and NP diagnosis is difficult, often leading to palliative rather than therapeutic protocols [7, 8].

Cognitive function assessment is one of the valuable clinical skills used to screen for cognitive impairment and assess the severity of a disease [9]. Many cognitive function assessment tools are available, including the Taiwan Mental State Examination [10], Montreal Cognitive Assessment [11], and AD8 Dementia Screening Interview [12]. However, the assessment of cognitive function with these tools is incomplete and insensitive to mild cognitive impairment due to the relative simplicity of these scales. Although a complete set of neuropsychological tests can comprehensively assess the level of cognitive function and the severity of cognitive impairment, these assessments generally take a long time, are prone to subject fatigue, and are especially unsuitable for the clinical evaluation of elderly individuals [13]. Therefore, tools that are simple to implement and can relatively comprehensively evaluate cognitive function have great application value. The Repeatable Battery for the Assessment of Neuropsychological Status (RBANS) overcomes the aforementioned shortcomings. It was originally used to identify pathological declines in cognitive function in the elderly population and to screen neuropsychological functional status in the general population [14]. To date, the RBANS has been applied to individuals with bipolar disorder [15], multiple sclerosis [16], cerebrovascular disease [17], epilepsy [18], and other diseases involving cognitive impairment. The results have shown that the RBANS could be used as an effective neuropsychological function screening tool, with good internal consistency, test-retest reliability, structural validity, and parallel validity, and sex had no significant effect on the evaluation results of the RBANS [19–21]. All these findings implied that the RBANS could be a favourable means of assessing cognitive function in patients with SLE.

Lipid metabolism abnormalities usually result in dyslipidaemia, which is prevalent in patients with SLE, with an incidence ranging between 18.1 and 75% [22]. Dyslipidaemia is characterized as disordered low-density lipoprotein (LDL) and/or decreased high-density lipoprotein (HDL) levels in the serum [23]. Dyslipidaemia is known to be associated with lupus disease activity, such as kidney damage and cardiovascular disease, which are the most common complications in patients with SLE and are closely related to the long-term prognosis [24]. Lipoprotein particles, such as chylomicrons, very low–density lipoproteins (VLDL), LDL and HDL, contain a class of apolipoproteins that play a crucial role in lipid metabolism. Apolipoproteins always serve as carrier, receptor-binding, and regulatory proteins in these particles to transport lipids between tissues for fuel and cholesterol metabolism and participate in the development of cardiovascular disease, obesity, diabetes mellitus and other diseases [25]. Regarding hippocampus-dependent cognition, researchers have found that a high-fat diet in rats impaired neurogenesis in the dentate gyrus of the hippocampus [26], decreased hippocampal production of brain-derived neurotrophic factor [27], increased apoptosis of hippocampal neurons, and was associated with a decreased weight of the hippocampus [28], which likely played a key role in neuronal loss, leading to learning and memory deficits. However, whether the NP manifestations in SLE are related to lipoproteins and their metabolism remains unclear. Nonthyroidal illness syndrome is also prevalent in SLE and characterized by decreased serum triiodothyronine (T3), thyroxine (T4), and thyroid-stimulating hormone levels [29–31]. Thyroid hormones can influence all aspects of lipid metabolism, including synthesis, mobilization, and degradation, thus resulting in dyslipidaemia [32]. Earliest descriptions of thyroid disease showed a link with neuropsychiatric disturbances, including significant psychiatric and cognitive disturbance, classical slowness of thought, and increased depressive symptoms [32]. Treatment with T4 has been shown to help improve disturbances in mood [33] but not help improve neurocognitive function, especially performance in complex attention tasks and verbal memory tests [34]. Inextricable links exist between SLE, dyslipidaemia and abnormal thyroid hormone levels. However, whether dyslipidaemia and thyroid hormone abnormalities in patients with lupus are related to cognitive dysfunction remains unknown.

In this study, correlations between cognitive function and levels of serum thyroid hormones (F-T3 and F-T4) and lipid metabolism-regulated molecules (apolipoprotein E (APOE) and apolipoprotein A1 (APOA1), insulin-like growth factor-1 (IGF-1) and insulin-like growth factor binding protein 7 (IGFBP7)) in SLE patients were evaluated. We aimed to discover changes in the levels of serum biomarkers and proteins possibly related to cognitive function in SLE that would allow the diagnosis or prediction of the prognosis of SLE.

## Materials and methods

### Patients and study design

A total of 121 patients with SLE who visited the Department of Rheumatology, Nanjing Drum Tower Hospital, Nanjing, China, from May 2019 to May 2020 were prospectively enrolled. All patients were diagnosed according to the SLE criteria of the American College of Rheumatology [35]. The disease activity of these patients was measured using the Systemic Lupus Erythematosus Disease Activity Index (SLEDAI) [36, 37]. Patients who had other autoimmune diseases; had a history of familial hyperlipidaemia and/or thyroid disease, diabetes mellitus, and/or other rheumatic diseases; and took lipid-lowering agents or thyroid medications were excluded. Active patients were classified into two groups based on the RBANS score: the low-cognition group (*n* = 33; RBANS score: 51–90) and the high-cognition group (*n* = 48; RBANS score: 91–130). Patients who developed neuropsychiatric syndromes not attributable to SLE (electrolyte imbalances, infections, or medications) were excluded. Meanwhile, 65 age- and sex-matched healthy controls (HCs) were recruited from the Physical Examination Center of Nanjing Drum Tower Hospital. This study was approved by the ethics committee at The Affiliated Drum Tower Hospital of Nanjing University Medical School (ID: SC201700201) and undertaken in accordance with the guidelines of the Declaration of Helsinki. At entry, patients completed a standardized medical history, laboratory tests, and analyses. All detections were carried out at the clinical laboratory of Nanjing Drum Tower Hospital. The demographic features of the patients and HCs are shown in Table 1.

**Table 1:** Demographic features of the 121 SLE patients and 65 healthy controls in the study

Variables	SLE (*n* = 121)	HCs (*n* = 65)	*P* Value
Age, (years)	33.88 (±11.77)	36.69 (±13.88)	0.150
Females, n (%)	106 (87.60%)	50 (76.92%)	0.093
Education, (years)	11.95 (±3.05)	13.28 (±4.58)	< 0.05*
SLEDAI	10 (0–47)	NA	/

Data are expressed as median (minimum - maximum, number (percentage), or mean ± standard deviation (SD) values. *P* values are based on independent sample t-test and Chi-square test for normally distributed variables. *P < 0.05, vs. HCs. The Graph Pad Prism 7.0 and SPSS Statistics 16.0 software were used for statistical analysis

### Cognitive dysfunction study

Cognitive dysfunction in SLE is characterized by deficits in attention, learning and recall, verbal and nonverbal fluency, language, visuospatial skills, executive functions, and motor dexterity [38–40]. A total of 121 patients with NPSLE and 65 normal volunteers were invited to participate in this study. All participants provided additional medical information using an ad hoc questionnaire and had a physical examination. Since the RBANS has been translated into a Chinese version and its clinical effectiveness and test-retest reliability have been established in healthy populations [41, 42], we applied the RBANS in our sample for cognitive function assessment. Generally, the RBANS contains twelve subtests that provide five general index scores and a total score. The test index scores included immediate memory (consisting of list learning and immediate story memory tasks), visuospatial/constructional (consisting of figure copy and line orientation tasks), language (consisting of picture naming and semantic fluency tasks), attention (consisting of digit span and coding tasks) and delayed memory (consisting of list recall, list recognition, figure recall, and delay story recall tasks); the detailed methods are provided in the supplementary materials. The stimuli were contained in a wire-bound, easel-type booklet, making the test easily portable and allowing for bedside administration. The total administration time was 20–30 min.

### Measurement of serum autoantibodies and lipid metabolism-related proteins

The levels of autoantibodies and lipoproteins (HDL-C and LDL-C) in the serum of patients were measured within 1 month of cognitive function testing. Thyroid hormones, including FT3, FT4 and TSH, were measured using a commercially available electrochemiluminescence (ECLIA) kit (Roche). The serum levels of APOE, APOA1, IGF-1 and IGFBP7 were detected using enzyme-linked immunosorbent assay (ELISA) kits following the manufacturer’s protocols [43]. The results were fitted to the standard curve, and the detection ranges were as follows: APOE (1.5–400 ng/mL), APOA1 (6.3–200 ng/mL), IGF-1 (62.5–4000 pg/mL), and IGFBP7 (625–40,000 pg/mL).

### Statistical analysis

Data were expressed as the means ± standard deviation. Differences between the two groups were determined using the unpaired-sample Student’s *t* test if the variance was normally distributed, and the Mann–Whitney *U* test was used for nonnormally distributed data. Differences among observed frequencies were tested using the chi-square test, while Pearson’s correlation coefficients were used to calculate the correlation between variables [43]. Additional correlations were calculated using Spearman’s correlation test. Data were analysed and visualized using SPSS 16.0 software or GraphPad Prism 7.0 (GraphPad Software, CA, USA), and a two-tailed *P* value < 0.05 was considered statistically significant.

## Results

### Clinical characteristics and cognitive function of patients with SLE and HCs

The demographic features of the 121 patients with SLE and 65 HCs in the study are summarized in Table 1. The mean ages of the patients and HCs were 33.88 ± 11.77 and 36.69 ± 13.88 years, respectively. The mean level of education was 11.95 ± 3.05 years in the patient group, which was significantly lower than that in the HC group (*P* <  0.01).

**Fig. 1:**
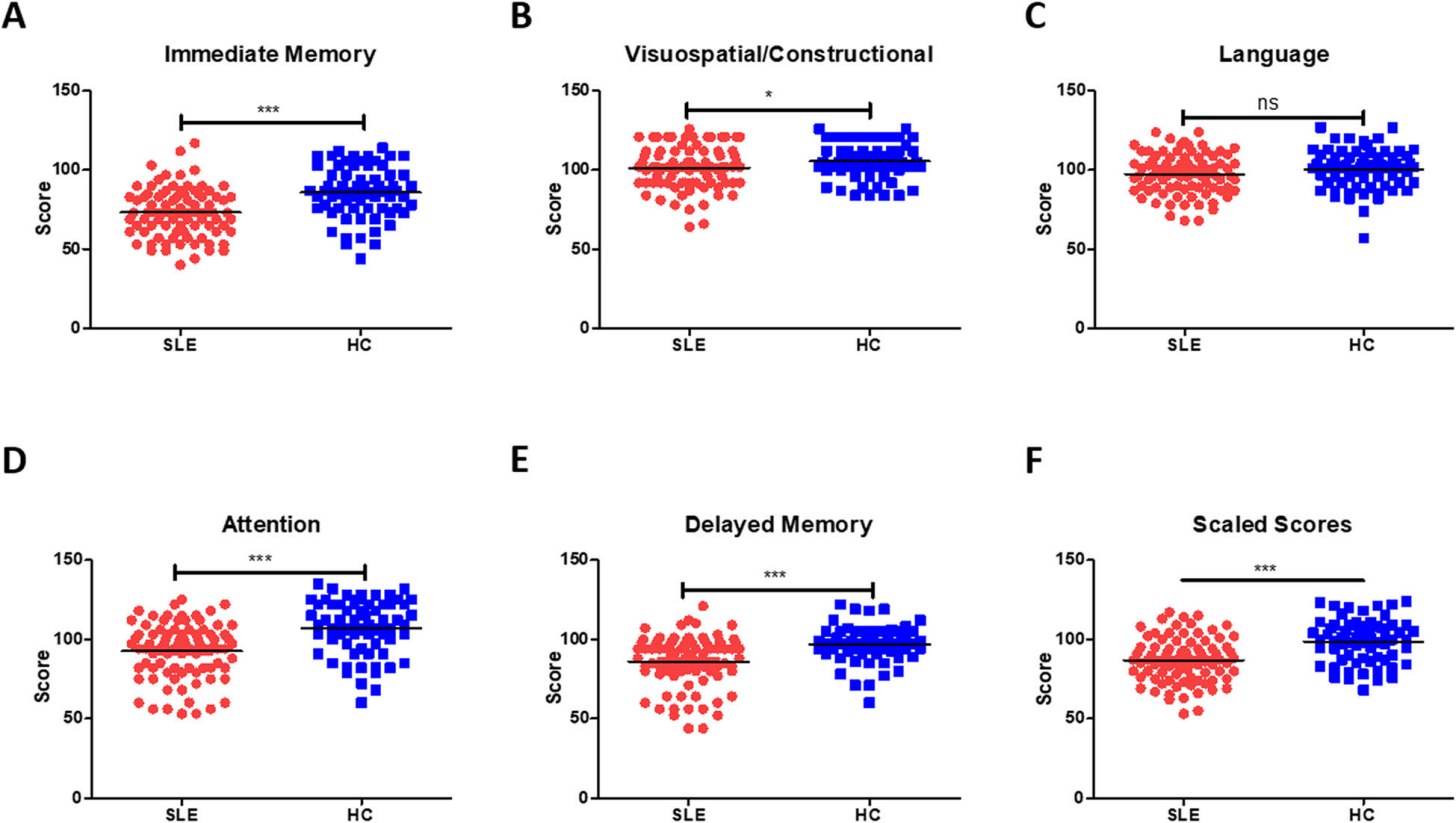
Cognitive function of patients with SLE and HCs. The differences in immediate memory (**a**), visuospatial/constructional (**b**), language (**c**), attention (**d**), and delayed memory (**e**) between patients with SLE and HCs. The scores in the above 5 cognitive areas were converted into scaled scores (**f**)

Afterward, the RBANS was performed on 121 patients and 65 HCs. Significant differences were found in list learning (*P* <  0.0001) and immediate story recall (*P* <  0.01) scores between the patients with SLE and HCs. The line orientation (*P* <  0.05) and picture naming (*P* <  0.01) scores were both lower in the SLE patients than in the HCs. In addition, the digit span (*P* <  0.05) and coding (*P* <  0.01) scores were lower in the SLE patients, as were the list recall (*P* <  0.01), list recognition test (*P* <  0.01), delayed story recall (*P* <  0.01), and figure recall (*P* <  0.05) scores. These tests corresponded to five cognitive domains: immediate memory, visuospatial/construction, attention, language, and delayed memory (Table 2). The results showed that levels of immediate memory, visuospatial/construction, attention, and delayed memory were significantly lower in the patients than in the HCs, while language features showed no obvious difference (Fig. 1).

**Table 2:** The data of commonly cognitive assessment

RBANS score	SLE (n = 121)	HCs (n = 65)	*P* Value
**Immediate memory**			
List Learning, median (range)	24 (5–39)	29 (15–36)	< 0.005***
Immediate Story Memory, median (range)	13 (1–22)	16 (1–21)	< 0.01**
**Visuospatial/constructional**			
Figure Copy, median (range)	20 (14–20)	20 (17–20)	0.2963
Line Orientation, median (range)	17 (8–20)	18 (9–20)	< 0.05*
**Language**			
Picture Naming, median (range)	9 (7–10)	9 (8–10)	< 0.01**
Semantic Fluency, median (range)	21 (7–33)	23 (11–35)	0.0873
**Attention**			
Digit Span, median (range)	13 (6–16)	14 (8–16)	< 0.05*
Coding, median (range)	46 (4–70)	54 (13–74)	< 0.01**
**Delayed memory**			
List Recall, median (range)	5 (0–10)	7 (2–10)	< 0.01**
List Recognition, median (range)	20 (16–20)	20 (17–20)	< 0.01**
Delay Story Recall, median (range)	7 (0–12)	9 (0–12)	< 0.01**
Figure Recall, median (range)	15 (5–20)	16 (7–20)	< 0.05*

Data are expressed as mean ± standard deviation (SD) values. *P* values are based on independent sample t-test for normally distributed variables. *P < 0.05, **P < 0.01, *** *P* < 0.005, vs. HCs. The Graph Pad Prism 7.0 software were used for statistical analysis

### Demographic features, clinical manifestations, and treatment of patients

Based on the RBANS scores, the 81 patients were divided into a high-cognition group (*n* = 33) and a low-cognition group (*n* = 48). As shown in Table 3, no statistically significant differences in age or sex were found between the two groups. The mean level of education was 10.77 ± 3.64 years in the low-cognition group, which was lower than that in the high-cognition group (*P* <  0.01). The levels of the SLEDAI (13.91 ± 11.79 vs 9.38 ± 5.79) were significantly higher in the patients with low cognition compared with those with high cognition (all *P* <  0.05). In addition, the disease duration between the two groups showed no difference (*P* = 0.2486).

**Table 3:** Demographic features, clinical manifestations, and treatment of SLE patients

SLE characteristics	Low Cognition (*n* = 48)	High Cognition (*n* = 33)	*P* Value
**Demographic**			
Age, (years)	34.67 (±12.03)	32.23 (±10.98)	0.3530
Females, n (%)	40 (83.33%)	30 (90.90%)	0.763
Education, (years)	10.77 (±3.64)	13.26 (±3.21)	< 0.01**
SLEDAI, mean ± (SD)	13.91 (±11.79)	9.38 (±5.79)	< 0.05*
Disease duration, (month)	61.01 (±59.69)	77.61 (±65.3)	0.2486
**Clinical chart review (%)**			
Rash, (%)	10 (21.28%)	6 (18.18%)	0.768
Mucosal ulcers, (%)	2 (4.26%)	1 (3.03%)	1.000
Haematuria, (%)	22 (46.81%)	13 (39.33%)	0.510
Proteinuria, (%)	22 (46.81%)	13 (39.33%)	0.510
Pyuria, (%)	14 (39.79%)	11 (33.33%)	0.736
Arthritis, (%)	8 (17.02%)	5 (15.15%)	0.823
Vasculitis, (%)	4 (8.51%)	0 (0%)	0.231
Pleurisy, (%)	1 (2.13%)	1 (3.03%)	1.00
Pericarditis, (%)	1 (2.13%)	0 (%)	1.00
Low complement, (%)	36 (76.60%)	27 (81.82%)	0.574
Anaemia, (%)	11 (23.40%)	1 (3.03%)	< 0.05*
Thrombocytopenia, (%)	11 (23.40%)	4 (12.12	0.203
Leukopenia, (%)	11 (23.40%)	7 (21.21%)	0.817
Lupus nephritis, (%)	19 (40.43%	20 (60.61%)	0.075
Neurological disorder, (%)	6 (12.77%)	5 (15.15%)	1.00
**Current medication (%)**			
Prednisone (%)	31 (64.58%)	18 (54.54%)	0.425
Hydroxychloroquine (%)	29 (61.70%)	19 (57.58%)	0.711
Cyclophosphamide (%)	5 (10.64%)	3 (9.09%)	1.000
Azathioprine (%)	1 (2.13%)	1 (3.03%)	1.000
Methotrexate (%)	2 (4.26%)	2 (6.06%)	1.000
Cyclosporine (%)	0 (0%)	2 (6.06%)	0.326

Data are expressed as number (percentage). *P* values are based on Chi-square test for normally distributed variables. *P < 0.05, **P < 0.01, vs. Low Cognition. The SPSS Statistics 16.0 software were used for statistical analysis

Patients with low cognition had more frequent anaemia (23.4% vs 3.03%) than patients with high cognition (*P* <  0.05). Meanwhile, the prevalence of rash, mucosal ulcers, haematuria, proteinuria, pyuria, arthritis, vasculitis, pleurisy, pericarditis, low complement, thrombocytopenia, leukopenia, lupus nephritis, and neurological disorder showed no difference between the two groups. In addition, the treatment with prednisone, hydroxychloroquine, cyclophosphamide, azathioprine, methotrexate, and cyclosporine was not different between the two groups (Table 3).

### Comparison of clinical and laboratory characteristics of patients

The study investigated the laboratory parameters of patients enrolled in this study (Table 4). The patients with low cognition had substantially lower albumin (31.76 ± 5.79 mg/dL vs 34.91 ± 4.13 mg/dL; *P* <  0.01), F-T3 (3.20 ± 1.03 pmol/L vs 3.71 ± 1.06 pmol/L; *P* <  0.05), and F-T4 (12.51 ± 3.43 pmol/L vs 14.97 ± 2.14 pmol/L; *P* <  0.05) levels than the patients with high cognition. The levels of anti-dsDNA antibody (*P* <  0.05) and IgM (*P* <  0.01) and the number of B cells (*P* <  0.05) were higher and the D-dimer levels were considerably higher in patients with low cognition (*P* <  0.001). The levels of lipid fractions, low-density lipoprotein cholesterol (LDL-C) and high-density lipoprotein cholesterol (HDL-C) displayed no differences between the two groups, and the other clinical indexes, including globulin, 24-h urine protein (24-h UP), C3, C4, C-reactive protein, and creatinine (Cr) showed no differences either.

**Table 4:** Comparison of clinical and laboratory characteristics of SLE patients

SLE characteristics	Low Cognition (n = 48)	High Cognition (n = 33)	*P* Value
**Clinical characteristics**			
Globulin, mg/dL	28.18 (±7.97)	27.94 (±6.59)	0.764
Albumin, mg/dL	31.76 (±5.79)	34.91 (±4.13)	< 0.01**
24-h urine protein, (g/24 h)	1.97 (±2.59)	1.88 (±3.11)	0.433
C3 Levels, mg/dL	0.66 (±0.32)	0.77 (±0.29)	0.143
C4 Levels, mg/dL	0.12 (±0.08)	0.14 (±0.06)	0.323
LDL-C (mmol/L)	2.77 (±1.37)	2.61 (±1.10)	0.590
HDL-C (mmol/L)	1.12 (±0.39)	1.33 (±0.55)	0.051
CRP (mg/dL)	12.36 (±14.00)	11.95 (±16.98)	0.869
D-dimer (mg/dL)	1.74 (±1.28)	0.75 (±0.82)	< 0.001***
Cr (μmol/L)	86.95 (±80.42)	66.07 (±36.13)	0.200
BUN (mmol/L)	8.04 (±5.66)	7.07 (±4.69)	0.491
UA (μmol/L)	362.20 (±129.73)	339.38 (±142.41)	0.523
GFR (mL/min/1.73 m^2^)	117.37 (±56.97)	140.31 (±83.36)	0.223
ESR (mm/hour)	50.53 (±32.03)	42.59 (±28.74)	0.271
PLT (10^9^/L)	149.32 (±79.69)	176.09 (±90.84)	0.099
TSH (mU/L)	2.78 (±2.31)	2.69 (±2.21)	0.830
FT3 Levels (pmol/L)	3.20 (±1.03)	3.71 (±1.06)	< 0.05*
FT4 Levels (pmol/L)	12.51 (±3.43)	14.97 (±2.14)	< 0.05*
**Blood Cells**			
Erythrocyte, (%)	68.66 (±118.01)	40.23 (±66.60)	0.419
WBC, (10^9^/L)	4.73 (±2.42)	5.43 (±3.62)	0.255
CD3+ cells, (%)	0.73 (±0.50)	0.67 (±0.40)	0.605
CD3 + CD4+ cells, (%)	0.33 (±0.22)	0.30 (±0.23)	0.591
CD3 + CD8+ cells, (%)	0.39 (±0.29)	0.36 (±0.19)	0.563
B cells, (%)	0.15 (±0.16)	0.10 (±0.13)	< 0.05*
NK cells, (%)	0.06 (±0.06)	0.06 (±0.04)	0.929
**Autoantibodies**			
Anti-dsDNA antibodies, U/mL	746.12 (±441.82)	314.56 (±223.99)	< 0.05*
Anti-Sm antibodies, n (%)	10 (21.28%)	8 (24.24%)	0.754
Anti-RNP antibodies, n (%)	16 (34.04%)	13 (39.39%)	0.624
Anti-SSA antibodies, n (%)	19 (40.43%)	14 (42.42%)	0.858
Anti-SSB antibodies, n (%)	4 (8.51%)	2 (6.06%)	1.000
Anti-Rib-P, n (%)	10 (21.28%)	11 (33.33%)	0.228
Anti-β2-GPI, n (%)	1 (2.13%)	0 (0%)	1.000
IgM (mg/dL)	1.13 (±0.72)	0.76 (±0.41)	< 0.01**
IgG (mg/dL)	14.52 (±6.79)	12.56 (±5.34)	0.172
IgA (mg/dL)	2.49 (±1.60)	2.56 (±1.19)	0.857
IgE (mg/dL)	0.26 (±0.44)	0.23 (±0.41)	0.620

Data are expressed as number (percentage), or mean ± standard deviation (SD) values. *P* values are based on independent sample t-test and Chi-square test for normally distributed variables. *P < 0.05, **P < 0.01, *** *P* < 0.005, vs. Low Cognition. The Graph Pad Prism 7.0 and SPSS Statistics 16.0 software were used for statistical analysis

In addition, differences in the proportion of various immune cells in the peripheral blood between the two groups was detected. The results showed that the number of B cells was higher in the low-cognition group (0.15 ± 0.16% vs 0.10 ± 0.13%; *P* <  0.05), but no difference was found in the numbers of erythrocytes, WBCs, CD3^+^ cells, CD3^+^CD4^+^ cells, CD3^+^CD8^+^ cells, and natural killer cells between the groups (Table 4). As shown in Table 4, the serum levels of anti-dsDNA antibodies (*P* <  0.05) and IgM (*P* <  0.01) were both higher in patients with low cognition. However, no statistically significant differences in the positive rates of anti-Sm antibodies, anti-RNP antibodies, anti-SSA antibodies, anti-SSB antibodies, anti-Rib P antibodies, and anti-β_2_GPI antibodies, as well as the levels of IgG, IgA, and IgE, were found between the two groups.

### Correlation analysis between cognitive function and F-T3, F-T4, and lipid metabolism-related protein levels

**Fig. 2:**
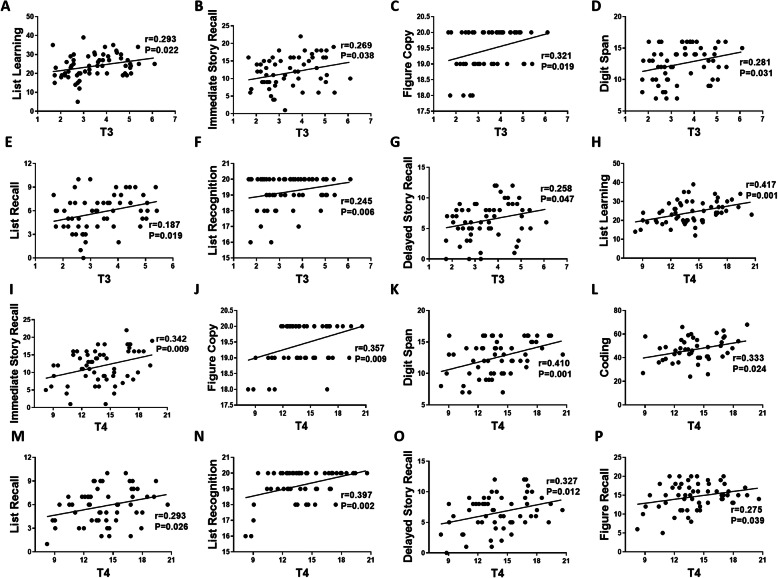
Correlation analysis between cognitive function and serum T3 and T4 levels. The correlations between T3 levels and list learning (**a**), immediate story recall (**b**), figure copy (**c**), digit span (**d**), list recall (**e**), list recognition (**f**), and delayed story recall (**g**) scores in patients with SLE. The correlations between T4 levels and list learning (**h**), immediate story recall (**i**), figure copy (**j**), digit span (**k**), coding (**l**), list recall (**m**), list recognition (**n**), delayed story recall (**o**), and figure recall (**p**) scores in patients with SLE

Studies have indicated that a significantly higher prevalence of thyroid autoantibodies has been observed in patients with SLE than in HCs [44, 45]. Many studies have attempted to associate thyroid abnormalities with clinical findings of SLE, but there has been no unified conclusion [46–48]. This study explored the relationships between serum F-T3 and F-T4 levels and cognitive functions in the patients with SLE. A regression analysis revealed that serum F-T3 levels positively correlated with immediate memory performance, including list learning (*r* = 0.293, *P* <  0.05; Fig. 2A) and immediate story recall (*r* = 0.269, *P* <  0.05; Fig. 2B). Moreover, figure copy (*r* = 0.321, *P* <  0.05; Fig. 2C), digit span (*r* = 0.285, *P* <  0.05; Fig. 2D), list recall (*r* = 0.187, *P* <  0.05; Fig. 2E), list recognition (*r* = 0.245, *P* <  0.01; Fig. 2F), and delayed story recall (*r* = 0.258, *P* <  0.05; Fig. 2G) scores also positively correlated with F-T3 levels. Serum F-T4 levels positively correlated with nine items (0.275 ≤ *r* ≤ 0.417; all *P* <  0.05; Fig. 2H–P), including list learning, immediate story recall, figure copy, digit span, coding, list recall, list recognition, delayed story recall, and figure recall scores.

**Fig. 3:**
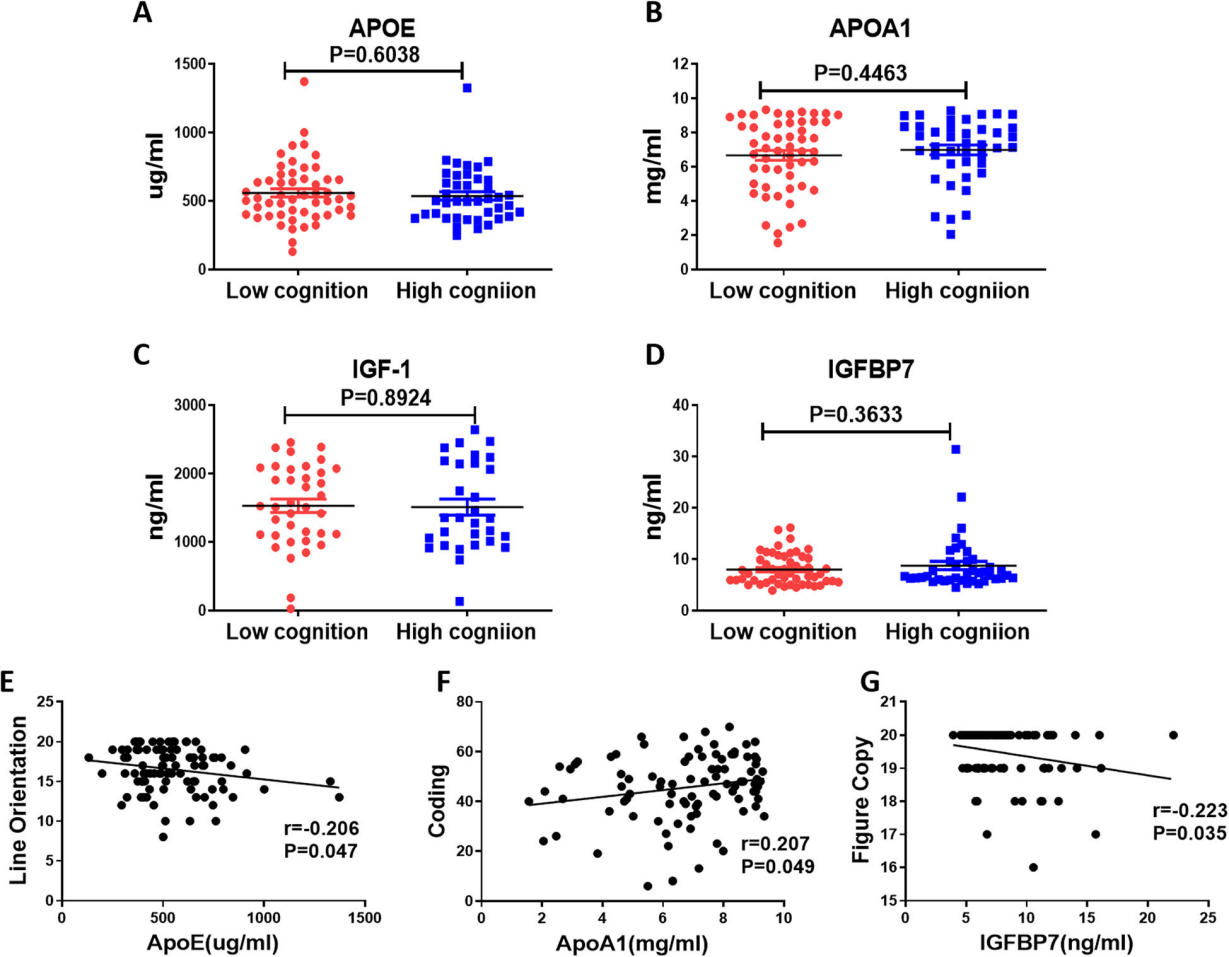
Expression of lipid metabolism-related proteins and correlations with cognitive function. The differences in serum APOE (**a**), APOA1 (**b**), IGF-1 (**c**), and IGFBP7 (**d**) levels between patients with SLE in different cognitive subgroups. The correlation between APOE levels and list orientation scores (**e**) in patients with SLE. The correlation between APOA1 levels and coding scores (**f**) in patients with SLE. The correlation between IGFBP7 levels and figure copy scores (**g**) in patients with SLE

The above results indicated that F-T3 and F-T4 levels positively correlated with most cognitive functions. Increasing evidence has shown that thyroid hormones (mainly T3 and T4) are involved in all aspects of lipid metabolism [49–51]. Moreover, lipid metabolism was markedly altered in patients with SLE [24]. We urgently wanted to know whether lipoproteins were related to cognitive dysfunction in SLE, and then we analysed the correlation between HDL-C and LDL-C and cognition. The results showed that HDL-C levels were only significantly positively correlated with immediate story memory (r = 0.081, *P* <  0.05) and delay story recall (r = 0.052, *P* <  0.05) scores, while LDL-C levels showed no correlation with any of the 12 cognitive subitems (Supplementary Fig. 1). In our previous study, we found that the plasma apolipoprotein (APOE and APOA1), IGF-1 and IGFBP7 levels were significantly different between NPSLE and SLE patients [52], implying that they might be related to neuropsychiatric manifestations. It is well known that apolipoproteins are involved in the composition and transportation of lipid particles [53]. We were surprised to find that IGF-1 and IGFBP7 could also regulate the abnormal metabolism of lipids [54, 55]. Then, the serum of 81 patients was collected for ELISA to detect the expression of the above four lipid metabolism-related proteins, and the correlations between lipid metabolism measures and cognitive dysfunction in patients with SLE was analysed. The results showed no significant differences in the expression of these four proteins between the two groups (Fig. 3A-D). We found that serum APOE levels significantly negatively correlated with line orientation scores (r = − 0.206, *P* <  0.05; Fig. 3E), whereas APOA1 levels showed a positive correlation with coding scores (r = − 0.207, *P* < 0.05; Fig. 3G). In addition, serum IGFBP7 levels negatively correlated with figure recall scores (r = − 0.223, *P* < 0.05; Fig. 3F), while IGF-1 levels showed no correlation with any cognitive measure. Considering that the characteristics of SLE are high levels of autoantibodies and damage to multiple organs [56], abnormal immunoglobulin levels have been observed in many patients [57–59]. We also analysed the correlations between cognitive function and serum levels of IgG, IgM and albumin. The results showed that IgG and IgM were both related to six subtests of cognition, while albumin correlated with five subtests of cognition (Supplementary Fig. 2).

## Discussion

In the present study, cognitive function was lower in patients with SLE than in HCs. Meanwhile, lower education, higher SLEDAI scores, lower albumin, F-T3, and F-T4 levels, and higher D-dimer, anti-dsDNA antibodies, and IgM levels were found in patients with low cognition than in those with high cognition. In addition, cognitive function was closely associated with the IgG, IgM, albumin, F-T3, and F-T4 levels in patients. In addition to these findings, APOE, APOA1, IGF-1 and IGFBP7 levels were detected by ELISA. Although the expression levels of these four proteins did not show any difference between the high- and low-cognition SLE patients, correlations between cognition and APOE, APOA1, and IGFBP7 levels have been revealed. An important finding of the present study was that the abnormal levels of serum levels of F-T3 and F-T4 and APOE, APOA1, and IGFBP7 might be associated with cognitive dysfunction in patients with SLE.

NP is a severe complication of SLE that results in severe neurodegenerative changes and threatens life [60]. A variety of neurological manifestations characterize NP, making the diagnosis of NPSLE a formidable challenge for rheumatologists [61, 62]. Patients with NPSLE have had multiple NP events, of which 91.2% affected the CNS, including cognitive dysfunction, headache, mood disorder, seizures, anxiety, and psychoses. The most common clinical manifestation has been cognitive dysfunction, which occurred in 42.1% of patients [63]. In addition, nervous system involvement was the initial presentation of SLE, while more than 50% of NP occurred within the first 5 years after the onset of SLE [63]. Such findings indicate that patients with SLE were likely to experience cognitive dysfunction before the diagnosis of NPSLE. Therefore, our study recruited 121 patients with SLE and 65 HCs for cognitive assessment using the RBANS. The results showed that before the diagnosis of NPSLE, the cognitive functions of patients with SLE, including immediate memory, visuospatial/constructional, attention, and delayed memory, were significantly lower than the cognitive functions of HCs. Compared with the HCs, the patients with SLE had a worse response in the lingual gyrus, which has been associated with visual attention, visual encoding/processing, and working memory [64, 65]. This finding might explain why patients with SLE performed worse on attention, memory, and visuospatial/constructional memory tasks.

SLE is a prototypic autoimmune disease caused by the loss of B cell tolerance, subsequent recognition of self-antigens and becoming autoreactive [66, 67]. In this study, the proportion of B cells did not unexpectedly increase in patients. Additionally, B cell hyperreactivity could induce an accumulation of autoreactive plasma cells [68] and secrete a variety of autoantibodies, including anti-dsDNA, anti-Sm, anti-Ro, anti-SSA, anti-SSB, anti-Rib-P, and anti-β2-GPI, to mediate the occurrence of SLE [69]. Compared with the high-cognition group, the levels of anti-dsDNA and IgM were clearly increased in the low-cognition group, but the levels of anti-Sm, anti-RNP, anti-SSA, anti-SSB, anti-Rib-P, anti-β2-GPI, IgG, IgA, and IgE all showed no differences. The cerebrospinal fluid (CSF)/serum quotient of albumin, known as quotient albumin (Q albumin), is widely accepted as a biomarker for estimating blood–brain barrier (BBB) function [70]; the BBB plays a critical role in the pathogenesis of NPSLE [71]. In the present study, the plasma albumin level, as a biomarker of disease activity in SLE [72], was lower in the low-cognition group. The results indicated that the changes in serum IgM, dsDNA, and albumin levels might be associated with cognitive dysfunction in patients with SLE. As expected, IgM levels showed negative correlations with cognitive function, albumin positively correlated with cognitive function, but dsDNA showed no correlation.

The association between thyroid disease and SLE was reported more than 50 years ago [73]. Both hypothyroidism and thyroid nodules are found more frequently in patients with SLE than in the general population [45]. In fact, the incidence rate of thyroid cancer is twice as prevalent in patients with SLE as in those without SLE [74]. In recent years, the impact of serum thyroxine on cognitive function has gradually attracted widespread attention, but no consistent conclusions have been drawn. A study on mild cognitive impairment and dementia showed that patients with relatively high T3 levels showed impairments in memory and visuospatial and executive functions [75]. However, patients with acute coronary syndrome and low T3 levels had a poorer health-related quality of life (including general health, social functioning, and role emotional) than those with normal levels at a 1-year follow-up [76]. A study on subjective cognitive decline showed that higher T3 levels were associated with better verbal memory performance (immediate and delayed recall tasks) in APOE ε4 carriers [77]. Our results showed that T3 and T4 levels were lower in patients with low cognition, and significant positive correlations were found between T3 and T4 levels and cognitive function, which was consistent with the aforementioned views and support the notion that T3 and T4 protected cognitive functions.

Dyslipidaemia is one of the major risk factors for SLE, leading to a high prevalence of premature atherosclerosis and coronary artery disease in patients with SLE [78, 79]. Impaired renal function is now known to be associated with dyslipidaemia [80, 81]. In addition, thyroid hormones influence all aspects of lipid metabolism; in particular, T3 induces LDL receptor gene expression and enhances LDL clearance [82]. A report also revealed that low free T3 levels were an independent risk factor for dyslipidaemia in patients with SLE [83]. Moreover, plasma lipoprotein metabolism is regulated and controlled by specific apolipoprotein constituents of the various lipoprotein classes [84, 85]. Apolipoproteins function in the regulation of lipoprotein metabolism through their involvement in the transport and redistribution of lipids among various cells and tissues, through their role as cofactors for enzymes of lipid metabolism, or through their maintenance of the structure of the lipoprotein particles [86]. An increasing number of studies have shown that apolipoproteins are involved in the occurrence of neuroinflammation. For example, APOE4 contributes to Alzheimer’s disease (AD) pathogenesis by modulating the metabolism, aggregation, and toxicity of amyloid-β peptide, tauopathy, synaptic plasticity, lipid transport, glucose metabolism, mitochondrial function, vascular integrity and so on [84, 87]. The present study did not show a difference in the expression of serum lipoproteins (i.e., APOE and APOA1) between the two groups, but the correlation analysis showed that APOE levels negatively correlated with line orientation scores and that APOA1 levels positively correlated with coding scores. Four years ago, an interesting result was reported: brain conditional APOE knockout (bKO) mice had synaptic loss and cognitive dysfunction, which was similar to that in total APOE KO mice, but (bKO) mice did not show the learning and memory impairment that had been observed in APOE KO mice [88]. These results indicated that the cognitive impairment in mice was specifically caused by systemic lipid metabolism. In AD, accelerated cognitive decline and an abnormal internal environment, structure, and function of the brain were also found in APOE ε4 carriers [89]. APOA1 is responsible for transporting cholesterol to the liver. It is a critical component in the formation of HDL. The overexpression of *APOA1* might effectively inhibit the age-related decline in memory and learning ability [90, 91]. Moreover, Kawano et al. found that the levels of APOA1 were strikingly lower in a group of late-onset nonfamilial AD [92]. These studies and our present research both suggested a positive correlation between APOA1 levels and cognitive functions. IGF-1 is an essential neurotrophic factor produced both peripherally and in the brain, and adequate levels of serum IGF-1 might be necessary for normal cognitive functioning [93, 94]. Serum IGF-1 levels were significantly elevated in patients with SLE and inversely correlated with age [95]. Shankar et al. supported the notion that both high and low levels of IGF-1 might induce poor cognitive function and that optimal levels of IGF-1 might be associated with better cognitive function [96]. However, in our study, IGF-1 did not differ between the high-cognition and low-cognition SLE patients, and there were no correlations between IGF-1 levels and 12 cognitive items. IGFBP7 is a protein belonging to the IGFBP superfamily that acts as a transporter of IGFs, lengthens their half-life, and regulates their access to their receptors [97]. In a new study in 2021, IGFBP7 was reported to be related to lipid metabolism in nonalcoholic fatty liver disease (NAFLD) [55]. In addition, it is also involved in the development of a variety of brain diseases [98–100]. In our results, IGFBP7 showed a negative correlation with figure copy scores, indicating that IGFBP7 might be related to visuospatial/constructional dysfunction in patients with SLE.

## Conclusions

In conclusion, the findings indicated that F-T3 and F-T4 levels were lower in patients with low cognition, and cognitive function was associated with T3, T4, APOE and APOA1 levels in patients with SLE. The results suggested that F-T3 and F-T4 could be used in clinical practice as biomarkers for cognitive dysfunction in patients with SLE.

## Supplementary Information

**Additional file 1.** The correlation analisis between cognitive function and HDL, IgG, IgM, Albumin seurm levels. **Supplementary Figure 1** Correlation analysis between cognitive function and HDL. Correlation between HDL-C and Immediate story recall (A) and delayed story recall (B) in patients with SLE. **Supplementary Figure 2** Correlation analysis between cognitive function and IgG, IgM, and albumin levels. Correlation analysis between cognitive function and IgG, IgM, and albumin levels.

## Data Availability

The datasets generated during and analyzed during the current study are not publicly available due to respect participants’ rights to privacy and to protect their identity, but are available from the corresponding author on reasonable request.

## Abbreviations

SLE: Systemic lupus erythematosus
NPSLE: Neuropsychiatric SLE
CNS: Central nervous system
LDL: Low-density lipoprotein
HDL: High-density lipoprotein
NTIS: No thyroidal illness syndrome
F-T3: Free triiodothyronine
F-T4: Free thyroxine
TSH: Thyroid-stimulating hormone
ACR: American College of Rheumatology
SLEDAI: SLE disease activity index
RBANS: Repeatable battery for the assessment of neuropsychological status
MMPI: Minnesota multiphasic personality inventory
CRP: C-reactive protein
Cr: Creatine
BUN: Blood urea nitrogen
UA: Uric acid
GFR: Glomerular filtration rate
ESR: Erythrocyte sedimentation rate
PLT: Platelets
TSH: Thyroid stimulating hormone
CSF: Cerebrospinal fluid
BBB: Blood–brain barrier
APOE: Apolipoprotein E
APOA1: Apolipoprotein A1
IGF-1: Insulin-Like growth factor-1
IGFBP7: Insulin-like growth factor binding protein 7
AD: Alzheimer’s disease
