# Supplementary material for: Association of lipoproteins and thyroid hormones with cognitive dysfunction in patients with systemic lupus erythematosus
Source: BMC Rheumatol. 2021 Jun 9;5:18. doi: 10.1186/s41927-021-00190-7 (PMC8188676; doi:10.1186/s41927-021-00190-7)
Supplement: Supplementary file 1 — Additional file 1. The correlation analisis between cognitive function and HDL, IgG, IgM, Albumin seurm levels. Supplementary Figure 1 Correlation analysis between cognitive function and HDL. Correlation between HDL-C and Immediate story recall (A) and delayed story recall (B) in patients with SLE. Supplementary Figure 2 Correlation analysis between cognitive function and IgG, IgM, and albumin levels. Correlation analysis between cognitive function and IgG, IgM, and albumin levels. [file 41927_2021_190_MOESM1_ESM.docx]

**Supplementary material**

**Association of lipoproteins and thyroid hormones with cognitive dysfunction in patients with systemic lupus erythematosus**

Li Lu^1,2,3#^, Wei Kong^1,2#^, Kangxing Zhou^1,2^, Jinglei Chen^1,3^, Yayi Hou^1,2,3*^, Huan Dou^1,2,3*^, Jun Liang^1,2*^

**1. Materials and Methods**

The RBANS consisted of five indexes: immediate memory, visuospatial/constructional, attention, language, and delayed memory [1]. Stimuli were contained in a wire-bound, easel-type booklet, making the test easily portable and allowing for bedside administration. The total administration time was 20–30 min. The battery indexes were assessed using the following tests [1]:

1. **List Learning:** The examiner read aloud a list of 10 words, which were semantically unrelated, early acquired, relatively high-imagery, and as phonetically unique as possible. The participants were asked to recall (without regard for order) as many of these items as possible. After four trials, the number of words correctly recalled on the fourth trial was used as a measure of verbal learning and memory; the total score was 40 points.
2. **Immediate Story Memory:** The examiner read a short story including 12 keywords aloud twice, and then the participants were asked to recall this story as possible. The number of keywords correctly recalled was used as a measure of immediate story memory to avoid complicated scoring rules; the total score was 24 points.
3. **Figure Copy:** The participants were asked to remember a geometric figure comprising 10 parts and then copy this figure in 4 min. Each part received a 2-point score (accuracy and placement); the total score was 20 points.
4. **Line Orientation:** Two lines were presented at an angle, and the participants needed to select the corresponding lines on a simultaneously presented array. The subject’s task on each item was to identify the matching lines. One point was given for each correctly matched line; the total score was 20 points.
5. **Picture Naming:** The participants were shown 10 pictures and asked to name the objects of the pictures. Semantic cues were given if the object was obviously misperceived (e.g., ‘‘umbrella’’ for mushroom). Naming correctly (including the correct answer according to the prompts) was used as a measure of picture naming; the total score was 10 points.
6. **Semantic Fluency:** The participants should generate the words as much as possible for a given semantic category (e.g., fruits and vegetables) within 60 s. The semantic categories aimed to minimize retrieval demands and thereby more specifically tap semantic stores rather than retrieval strategies. One point was given for each correct word; the total score was 20 points.
7. **Digit Span:** The Digit Span was analogous to digits forward on the Wechsler Adult Intelligence Scale (WAIS). There were two string of digits in each item, increasing from 2 to 9 digits. The second given length string was read when the first string was failed. The failure of attempts of two same items was given 0 points; the total score was 16 points.
8. **Coding:** First, the participants were provided with a row of graphics, each of which corresponded to a number. Later, they were provided with some figures without numbers. They were asked to fill in the number under each figure provided for the second time as fast as possible, according to the corresponding relationship between the figure and the number provided for the first time. Numbers rather than symbols were chosen for the response to avoid the possible detrimental effect of constructional apraxia on performance. The score was the total number of items completed in 90 s.
9. **List Recall:** In this test, the participants were asked to recall the words provided in the “List Learning” test as many as possible. One point was given for each correctly recalled words, and the total score was 16 points.
10. **List Recognition:** In this test, the examiner read 20 words aloud, including 10 words provided in the "List Learning" test. The participants were asked to answer whether the words were read before. One point was given for each correct recognition, and the total score was 20 points.
11. **Delay Story Recall:** In this test, the participants were asked to recall the story provided in the “Story Memory” test. The number of keywords correctly recalled was used as a measure of delay story memory to avoid complicated scoring rules. One point was given for each correctly recalled keyword, and the total score was 12 points.
12. **Figure Recall:** In this test, the participants were asked to recall and draw the figure provided in the “Figure Copy” test as accurate as possible. Each part received a 2-point score (accuracy and placement) for 20 possible points.

SCALING: For the purpose of the present study, a single reference sample was used to provide the scaling metrics. The norm was established based on the test results of 540 people aged 20–89 years. Raw scores were converted into scaled scores for all participants in the present study using the same scaling metric, regardless of age [1-2].

**2. Results**

**Supplementary Figure 1 Correlation analysis between cognitive function and HDL.**


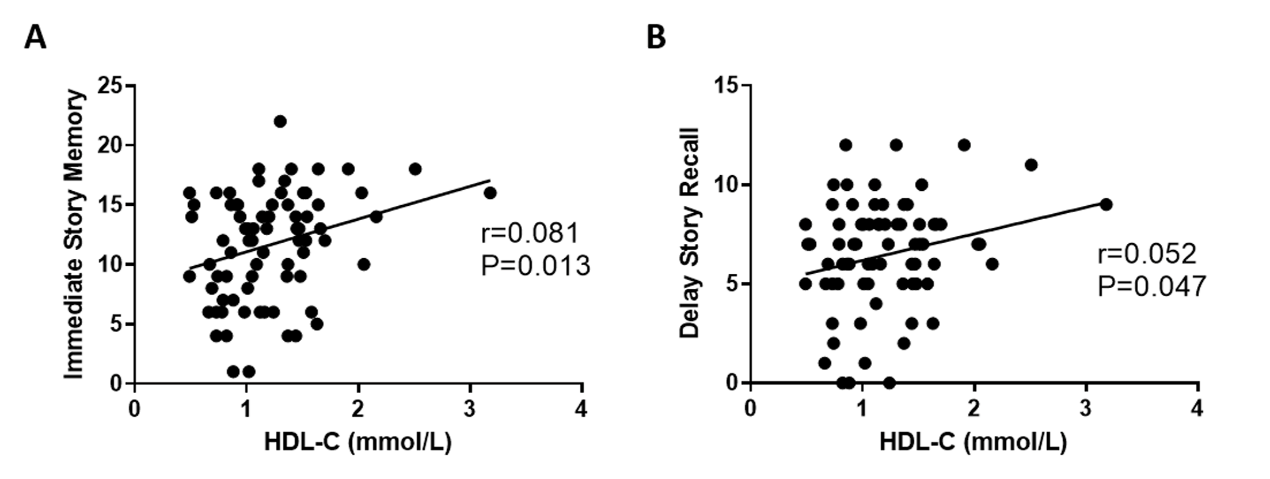


**Supplementary Figure 1**

Correlation between HDL-C and Immediate story recall (A) and delayed story recall (B) in patients with SLE.

**Supplementary Figure 2 Correlation analysis between cognitive function and IgG, IgM, and albumin levels**


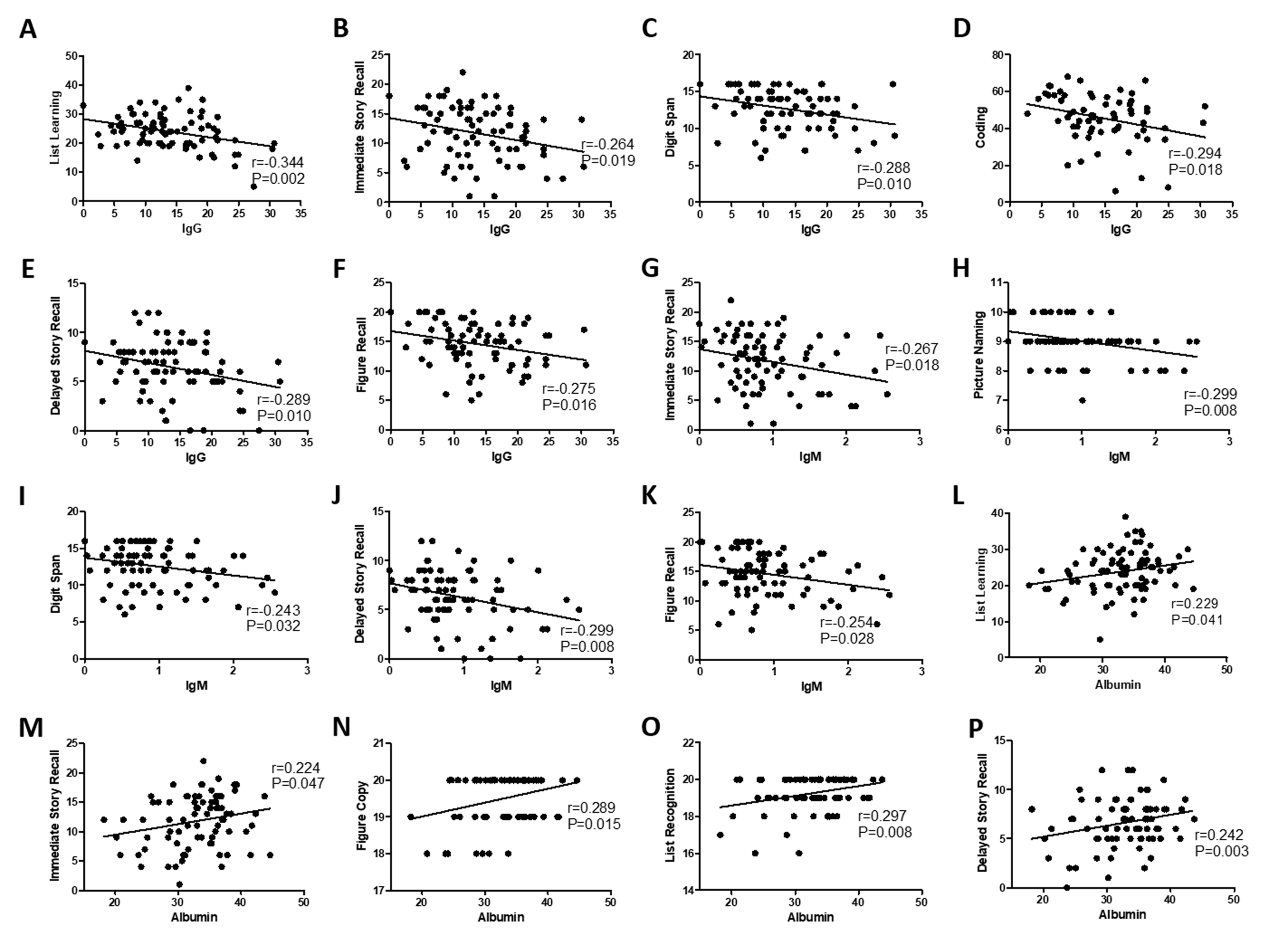


**Supplementary Figure 2 Correlation analysis between cognitive function and IgG, IgM, and albumin levels**

Correlation between IgG and list learning (A), immediate story recall (B), digit span (C), coding(D), delayed story recall (E), and figure recall (F) in patients with SLE.

Correlation between IgM and Immediate story recall (G), picture naming (H), digit span (I), delayed story recall (J), and figure recall (K) in patients with SLE.

Correlation between IgM and list learning (L), immediate story recall (M), figure copy (N), and list recognition (O) in patients with SLE.

The results showed that the IgG level was inversely associated with neuropsychological tests of immediate memory (list learning: *r* = –0.344, P < 0.01; immediate story recall: *r* = –0.264, *P* < 0.05; Supplementary Fig. 1A and 1B). Besides, attention (digit span: *r* = –0.288, *P* < 0.0*1*; coding: *r* = –0.294, *P* < 0.05; Supplementary Fig. 1C and 1D) and delayed memory (delayed story recall: *r* = –0.289, *P* < 0.01; figure recall: *r* = –0.275, *P* < 0.05; Supplementary Fig. 1E and 1F) showed a similar correlation. The serum IgM level showed a significant negative correlation with immediate story recall (*r* = –0.267, *P* < 0.05; Supplementary Fig. 1G), picture naming (*r* = –0.299, *P* < 0.01; Supplementary Fig. 1H), digit span (*r* = –0.243, *P* < 0.05; Supplementary Fig. 1I), delayed story recall (*r* = –0.299, *P* < 0.01; Supplementary Fig. 1J), and figure recall (*r* = –0.254, *P* < 0.05; Supplementary Fig. 1K).

The results showed that the albumin levels correlated positively with list learning, immediate story recall, and figure copy (all P < 0.05; Supplementary Fig. 1L, M, and 1N), as well as list recognition test and delayed story recall (both P < 0.01; Supplementary Fig. 1O and 1P).

**Reference:**

[1] Christopher Randolph , Michael C. Tierney , Erich Mohr & Thomas N. Chase. The Repeatable Battery for the Assessment of Neuropsychological Status (RBANS): Preliminary Clinical Validity. J Clin Exp Neuropsychol. 1998 Jun;20(3):310-9.

[2] Cheng Yan, Li Chunbo, Wu Wenyuan. Application evaluation of a repeatable set of neuropsychological status tests. Chin J Psychiatry. February 2010, Vol. 43, No. 1 (in Chinese)
